# Numerical study of gene electrotransfer efficiency based on electroporation volume and electrophoretic movement of plasmid DNA

**DOI:** 10.1186/s12938-018-0515-3

**Published:** 2018-06-18

**Authors:** Tadeja Forjanič, Damijan Miklavčič

**Affiliations:** Faculty of Electrical Engineering, University of Ljubljana, Trzaska 25, 1000 Ljubljana, Slovenia

**Keywords:** Skin electroporation, Gene electrotransfer, Numerical modeling

## Abstract

**Background:**

The efficiency of gene electrotransfer, an electroporation-based method for delivery of pDNA into target tissues, depends on several processes. The method relies on application of electric pulses with appropriate amplitude and pulse duration. A careful choice of electric pulse parameters is required to obtain the appropriate electric field distribution, which not only controls the electroporated volume, but also affects the movement of pDNA. We used numerical modeling to assess the influence of different types of electrodes and pulse parameters on reversibly electroporated volume and on the extent of pDNA–membrane interaction, which is necessary for successful gene electrotransfer.

**Methods:**

A 3D geometry was built representing the mice skin tissue and intradermally injected plasmid volume. The geometry of three different types of electrodes (plate, finger, needle) was built according to the configuration and placement of electrodes used in previously reported in vivo experiments of gene electrotransfer. Electric field distribution, resulting from different pulse protocols was determined, which served for calculation of reversible electroporation volume and for simulation of electrophoretic movement of pDNA. The efficiency of gene electrotransfer was evaluated in terms of predicted amount of pDNA present inside the volume of reversible electroporation at the end of pulse delivery.

**Results:**

According to results of our numerical study, finger and needle electrodes provide larger amount of pDNA inside the volume of reversible electroporation than plate electrodes. However, these results are not consistent with the experiments showing that plate electrodes achieve the best transfection efficiency. Some inconsistencies were observed also by comparing the efficiencies of different high and low voltage pulse combinations, delivered by plate electrodes. The reason for inconsistencies probably lies in insufficient knowledge regarding the electroporation of stratum corneum. Namely, the size of the regions with high electrical conductivity, created by electroporation, was found to strongly affect predicted transfection efficiency.

**Conclusions:**

The presented numerical model simulates the two most important processes involved in gene electrotransfer: electroporation of cells, and electrophoretic movement of pDNA. The inconsistencies between the model and experiments indicate incomplete knowledge of skin electroporation, or the involvement of other mechanisms, whose importance has not been yet identified.

## Background

Low permeability of the cell membrane represents a major barrier to successful gene transfer. One of the physical methods that can be employed to overcome this barrier is based on cell membrane electroporation. The method, termed gene electrotransfer, relies on the delivery of electric pulses with appropriate amplitude, duration and repetition frequency, which results in increased permeability of the cell membrane, a phenomenon known as electroporation [1, 2]. In addition to increasing the cell membrane permeability, electric pulses play an important role in transporting of pDNA towards cell membrane. Negatively charged DNA molecules move under the influence of electrophoretic force, generated by an external electric field [3, 4]. Due to the action of electrophoretic force, DNA molecules are brought in contact with more cells compared to free diffusion, thus increasing the probability of DNA-membrane complex formation, which are necessary for successful gene electrotransfer [4–6].

Therefore, electric pulses intended for gene electrotransfer have to be optimized in terms of electroporation volume and in terms of DNA-membrane interaction, while preserving cell viability [7]. As shown in several studies in vivo and in vitro [8–11], the best transfection efficiency is achieved by a combination of short high voltage (HV) and long low voltage (LV) pulses. This is not surprising, since the use of different pulses enables to separately control both factors of gene electrotransfer efficiency—HV pulses control the electroporation volume and LV pulses control the extent of pDNA–membrane interaction.

Optimization of pulse parameters for gene electrotransfer into the skin has been performed mainly experimentally [12]. So far, a few numerical models have been developed to investigate the gene electrotransfer efficiency in terms of electric field distribution [13–15] and Joule heating [16]. In this paper, we present a numerical model including additional parameter of gene electrotransfer efficiency-distribution of plasmid DNA due to action of electrophoretic force. More specifically, we were interested in the amount of plasmid DNA inside the volume of reversible electroporation depending on the electrode configuration (plate, finger, needle) and pulse parameters used. The predictive power of the model was tested on experiments published by Calvet et al. [17].

## Methods

**Fig. 1:**
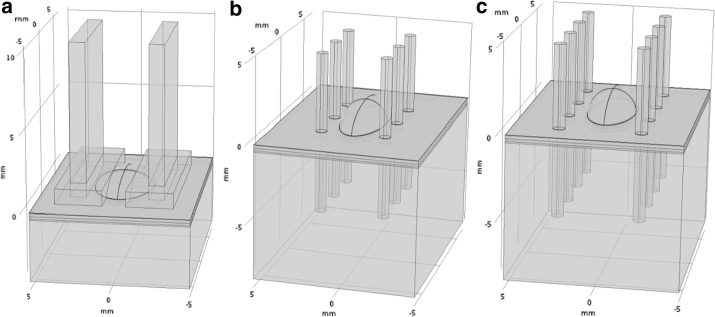
Geometry used in numerical modeling representing the skin tissue, injected plasmid volume and configuration of the following electrodes: **a** plate, **b** finger and **c** needle electrodes

A three dimensional numerical model was developed to simulate the experiments of Calvet et al. [17]. Three geometries were built considering the configuration of three different types of electrodes used in experiments—finger, needle and plate (Fig. 1). Tips of needle and finger electrodes were positioned 5 mm below the skin surface. The geometry of the skin consisted of the following layers: stratum corneum, epidermis, dermis, adipose tissue, muscle tissue and subcutaneous tissue (Table 1). An ellipsoid with the volume of 25 mm^3^ was placed in the middle of the dermis representing the intradermally injected plasmid volume. Since electrodes were placed in such a way they surrounded the bleb formed by the plasmid volume and were in contact with it [17], we adjusted the dimensions of the ellipsoid for each electrode configuration. In the case of plate electrodes, for instance, the size of ellipsoid in the direction perpendicular to the electrodes was the largest, 1.8 mm. Finger and needle electrodes had narrower gap between both rows of electrodes, therefore, the corresponding size of the bleb was reduced to 1.3 mm in order to fit between the electrodes (Fig. 1).

**Table 1:** Definition of numerical model: geometry of the skin together with electrical and thermal properties of the skin tissue, plasmid volume and electrodes

	Thickness (μm)	Electrical conductivity (S/m)	Density (kg/m^3^)	Thermal conductivity (W/m K)	Heat capacity (J/kg K)
Local transport region (LTR)	5	0.1	1400	0.2	3600
Stratum corneum	5	0.0001	1400	0.2	3600
Epidermis	15	0.2–0.8^a^	1200	0.24	3600
Dermis	200	0.2–0.8^a^	1200	0.45	3300
Adipose tissue	150	0.05–0.2^a^	900	0.19	2400
Muscle tissue	90	0.5	1040	0.5	3350
Subcutaneous tissue	2000	0.05	900	0.19	2400
Plasmid volume	–	1.4	1000	0.6	3600
Electrodes	–	1.35 × 106	7810	16.9	477

^a^Nonpermeabilized—fully permeabilized tissue

A steady-state Laplace equation was employed to calculate electric field distribution:

1 $$ \nabla \cdot \left( {\sigma \left( E \right) \nabla \varphi } \right) = 0, $$

where $\sigma$ is the electrical conductivity (Table 1) and $\varphi$ is the electric potential. All boundaries of the geometry, except for electrodes, were treated as electrically insulated. In the case of plate electrodes, the applied voltage amplitude (*φ*_0_) was prescribed as a boundary condition at the surface of one of the electrodes (*φ* = *φ*_0_). The surface of the other electrode was set to ground ($\varphi = 0 \,{\text{V}}$). Similar boundary conditions were assigned to needle and finger electrodes—one row of electrodes was set to applied voltage, while the other row was set to ground.

When pulses are delivered with plate electrodes, electrical properties of stratum corneum dictate the electroporation of underlying skin layers. The high resistance of stratum corneum begins to drop after exceeding electroporation threshold [18]. The reduced resistance is related to the formation of small regions with high electrical conductivity, so called local transport regions (LTRs) [19]. Small cylinders were introduced in the geometry to simulate the LTRs at the beginning of the HV pulse. Cylinders were distributed throughout the area of stratum corneum beneath the gel, which was applied between the electrodes and skin to improve the contact. Two different initial diameters of LTRs were used in simulation to investigate the effect of stratum corneum conductivity—10 and 20 µm. The density of LTRs, which enable the electroporation of underlying tissue, increases with the pulse amplitude. In the model, we used the density of 60 LTRs per 0.1 cm^2^, which lies in the middle of the reported range of LTR densities [20]. The size of LTRs increases during the pulse delivery due to lipid melting caused by Joule heating. The phase transition of stratum corneum lipids occurs at around 70 °C. In the numerical model, stratum corneum was assumed to undergo an irreversible phase transition locally in the LTR in the temperature range between 65 and 75 °C with the latent heat of 5300 J/kg [21]. Since finger and needle electrodes penetrate into the skin, the impact of stratum corneum on electric field distribution is decreased with respect to plate electrodes. Except for the stratum corneum, which was treated as a bulk layer without LTRs, electrical properties of other layers were the same as in the case of plate electrodes.

The electric field amplitude required to achieve electroporation, decreases with the duration of pulses in a strongly nonlinear fashion [22]. For pulses shorter than about 1 ms, threshold electric field decreases sharply with pulse duration, while for longer pulses (above 1 ms), this decrease becomes progressively smaller. The reversible and irreversible electroporation threshold of the skin for 100 µs pulse (600 and 1200 V/cm, respectively) were taken from literature [23]. To determine both thresholds for LV pulse, we selected the best two fits describing the relation between electric field and pulse duration from [24]. For 400 ms long pulse, the average electric field for electroporation was six times lower than for 100 µs pulse. Therefore, the reversible and irreversible threshold for the LV pulse were set to 100 and 200 V/cm, respectively. Between both thresholds, electrical conductivity increases due to electroporation. The increase in conductivity with respect to electric field was assumed to follow a sigmoid curve [25, 26].

Temperature is another important variable to be considered in the optimization of gene electrotransfer parameters. Resistive heating leads to substantial temperature increase inside the LTRs, which can damage the surrounding cells. However, thermal damage affects smaller volume of cells than irreversible electroporation [27, 28] and was, therefore, not specifically evaluated. Also, all thermally damaged cells lie within the volume subjected to irreversible electroporation. Nevertheless, resistive heating was included in simulation, since it affects the expansion of LTRs. The resistive heat, generated during the pulses, was used as a source term in the heat transfer equation:

2 $$ \rho c\frac{\partial T}{\partial t} = \nabla \cdot \left( {k\nabla T} \right) + \sigma \left| {\nabla \varphi } \right|^{2} $$

*ρ*, *c* and *k* are mass density, specific heat capacity and thermal conductivity of the material, respectively. A stationary study was employed to calculate electric field distribution, since a time-dependent study considering the conductivity changes as a result of LTR expansion during the pulses would be computationally too demanding. The size of LTRs was updated before the calculation of electric field generated by the LV pulse. It turned out that neglecting the effect of LTR expansion during the pulse was justified also for long LV pulses. Namely, only the LV pulse with the highest voltage-to-distance ratio, 180 V/cm, produced sufficient heating for LTR expansion to occur.

### Gene electrotransfer modeling

We modeled the distribution of plasmid DNA in the skin tissue as a result of electrophoretic movement of plasmid DNA during the pulse delivery. Namely, negatively charged pDNA molecules migrate toward the anode under the influence of electrophoretic force, generated by local electric field. In addition to the local electric field E, the distance L travelled by DNA molecules depends on their mobility (*μ*) and the duration of pulses (*τ*) [29]:

3 $$ L = \mu  E \tau $$

In [29], the mobility of 1.5 × 10^4^ µm^2^/Vs is estimated for a plasmid with 4700 base pairs. Since electrophoretic mobility is proportional to the length of the plasmid, the following values were used in simulations: *μ* = 2.0 × 10^4^ µm^2^/Vs for pCMV-luc plasmid with 6233 base pairs and *μ* = 2.3 × 10^4^ µm^2^/Vs for INVAC-1 plasmid with 7120 base pairs.

Charged particle tracing module provided by Comsol Multiphysics^®^ (v5.3, Stockholm, Sweden) was employed to simulate the trajectories of DNA molecules. We neglected the contribution of HV pulse to electrophoresis due to its short duration (100 μs). Therefore, the simulation of trajectories was based on the stationary electric field distribution, generated by the LV pulse. Charged particles were released from the surface of ellipsoid to model the flux of DNA molecules from the plasmid volume to the skin tissue. Particles were released uniformly across the surface of the ellipsoid every 10 ms to simulate the continuous inlet of DNA molecules into the skin tissue during the pulse delivery. After determining the distribution of particles at the end of the LV pulse, we assessed the number of particles present inside the volume of reversible electroporation as a measure of transfection efficiency. The time step of 10 ms between particle releases was short enough not to affect the results in terms of relative transfection efficiencies. Also, increasing the number of simulated particles or increasing the density of the mesh did not affect relative transfection efficiencies.

## Results

In the study of Calvet et al. [17] it was found that plate electrodes are more suitable for electrotransfer of pCMV-luc and INVAC-1 plasmids than both invasive electrodes (finger and needle). The difference between invasive and noninvasive electrodes was more pronounced in the case of INVAC-1 plasmid. Interestingly, the numerical model predicts a better gene electrotransfer efficiency of invasive electrodes (Table 2). According to the model, invasive electrodes should be more suitable due to significantly larger volume of reversible electroporation. However, the volume of reversible electroporation in the case of plate electrodes strongly depends on the electrical properties of stratum corneum, which are not completely understood. Particularly important for electroporation of underlying layers are the size and the distribution of LTRs. As we can see from Table 2, increasing the initial size of LTRs from 10 to 20 µm in diameter resulted in more than 30% larger volume of reversible electroporation. Consequently, similar increase is obtained in amount of plasmid DNA brought in contact with electropermeabilized cells, ranging from 28% in the case of INVAC plasmid to 37% in the case of pCMV-luc plasmid. It is important to emphasize that reversible electroporation volume was evaluated at the end of the HV pulse, taking into account the expansion of LTRs during the pulse. The diameter of LTRs increased, on average, for 10 μm regardless of the initial size of LTRs.

**Table 2:** Model-based prediction of gene electrotransfer efficiency resulting from the pulse combination 1000 V/cm (100 μs) + 140 V/cm (400 ms)

	Plate (LTR diameter = 10 μm)	Plate (LTR diameter = 20 μm)	Finger	Needle
Volume of reversible electroporation—dermis (mm^3^)	2.61	3.61	6.18	10.19
Volume of reversible electroporation—all layers (mm^3^)	16.36	21.63	216.40	330.72
pCMV-luc (6233 bp)				
Number of charged particles inside the volume of reversible electroporation—dermis	307	422	1024	937
Number of charged particles inside the volume of reversible electroporation—all layers	310	422	1205	994
INVAC-1 (7120 bp)				
Number of charged particles inside the volume of reversible electroporation—dermis	305	387	941	870
Number of charged particles inside the volume of reversible electroporation—all layers	305	389	1122	931

The measure of gene electrotransfer efficiency is the number of charged particles representing plasmid DNA inside the volume of reversible electroporation at the end of the pulse delivery. From all particles released from the surface of the plasmid volume, 5000 were selected randomly for evaluation. Increasing the number of evaluated particles did not affect the relative gene electrotransfer efficiencies

Comparison of different electrodes for gene electrotransfer was followed by a comparison of various HV–LV pulse combinations, delivered by plate electrodes (Table 3). In experiments, the best pulse combinations in terms of luciferase expression proved to be 1000 V/cm + 180 V/cm, 1400 V/cm + 140 V/cm and 1400 V/cm + 180 V/cm. These three pulse combinations, in addition to 1000 V/cm + 140 V/cm pulse combination, were then tested for vaccination with INVAC-1. Both pulse combinations with the amplitude of HV pulse being 1400 V/cm achieved higher transgene expression than pulse combinations with lower HV pulse amplitude (1000 V/cm).

**Table 3:** Gene electrotransfer efficiencies of different HV–LV pulse combination, delivered by plate electrodes

HV pulse amplitude (V/cm)	LV pulse amplitude			
	60 V/cm	100 V/cm	140 V/cm	180 V/cm
1000	316	305	310	337
1400	441	427	455	552

The initial diameter of LTRs was 10 μm. The electrophoretic movement of charged particle during the HV pulse was neglected due to its short duration (100 μs). The values in the table represent the number of charged particles inside the volume of reversible electroporation at the end of the LV pulse out of 5000 randomly selected particles. Reversible electroporation volume was determined from the HV pulse, except for the 1000 V/cm + 180 V/cm pulse combination, where LV pulse generated larger volume of reversible electroporation than HV pulse

According to numerical model, the pulse combination 1000 V/cm + 180 V/cm is the only one where LV pulse generates larger volume of reversible electroporation (10.1 mm^3^) than HV pulse (9.1 mm^3^). The same LV pulse (180 V/cm) is also the only one that generates sufficient heating for LTR expansion. Nevertheless, the diameter of LTRs does not increase for more than 10 μm.

The impact of the HV pulse intensity on the predicted amount of DNA inside the volume of reversible electroporation is as expected-higher amplitude of the pulse leads to larger volume of reversible electroporation and, therefore, more DNA molecules come in contact with permeabilized cells (Table 3). However, the importance of HV pulse amplitude was indicated only in terms of INVAC-1 expression. Luciferase expression, on the other hand, showed only moderate dependency on the HV pulse amplitude. Instead, the gradual increase in luciferase expression was achieved by increasing the amplitude of LV pulses. This is not consistent with results of numerical modeling, which predict more pronounced effect of HV than LV amplitude on gene expression. Namely, the number of charged particles inside the volume of reversible electroporation depends more strongly on the HV amplitude than LV amplitude (Table 3). The inconsistencies between the model and experiments show insufficient accuracy of electroporation model and/or incomplete knowledge and understanding of parameters determining gene electrotransfer efficiency.

## Discussion

Several parameters affecting gene electrotransfer efficiency have been identified so far [7, 30, 31]: electric field distribution, which is related to the electrode configuration and pulse parameters; electrophoretic movement of the plasmid; the delivery site [32]; pH changes near the electrodes [33]; the design of plasmid and plasmid concentration [34]. The role and importance of these factors need to be established to achieve consistent and high efficiency of gene electrotransfer. In this study, we used numerical modeling as an alternative tool to assess the influence of the first two parameters: electric field distribution and electrophoretic movement of the plasmid.

The first parameter considered was the electric field distribution, which depends on electrode geometry and placement. As expected, both invasive electrodes used (i.e. finger and needle) achieved larger volume of reversible electroporation, since they penetrate through stratum corneum. Therefore, the insulating properties of stratum corneum do not hinder the electroporation of underlaying layers. Based on results of our model, the electroporation of skin layers other than dermis is less significant, since majority of plasmid DNA does not reach the adjacent layers. Interestingly, although needle electrodes generate larger volume of reversible electroporation than finger electrodes, the predicted amount of plasmid DNA contributing to electrotransfer efficiency is smaller. This apparent discrepancy can be explained by stronger electrophoretic force generated by finger electrodes, which pulls more DNA molecules inside the reversible electroporation volume. However, the experiments by Calvet et al. [17] do not fully confirm these predictions, since finger electrodes proved to be a better choice only in the case of INVAC-1 plasmid. Namely, needle electrodes performed better than finger when using pCMV-luc plasmid to evaluate the transfection efficiency.

Even larger discrepancy is observed when comparing finger and needle electrodes to plate electrodes. Regardless of the plasmid used in experiments, plate electrodes achieved the highest transfection efficiency, contrary to results predicted by numerical modeling. One of the reasons for this discrepancy may lie in incomplete understanding of the conductivity changes of the stratum corneum due to electroporation. The conductivity of the stratum corneum namely strongly depends on the size and the density of structural alterations (LTRs) caused by electric pulses. It is known that the density of LTRs associated with HV pulses is higher, but their size is smaller compared to LTRs associated with LV pulses. However, more precise knowledge about this relationship is required, since the size and the distribution of LTRs after the HV pulse strongly affects the evolution of LTRs during subsequent LV pulse. If their size is too small or their density is too high, there is no sufficient Joule heating for phase transition of lipids (i.e. LTR expansion) [35]. Hence, the conductivity of stratum corneum does not increase and fewer cells experience reversible electroporation. For our particular geometries with the initial diameters of LTRs being 10 and 20 µm, no thermal expansion of LTRs was obtained during the LV pulse with the amplitude of 140 V/cm. As expected, the geometry with larger size of LTRs (20 µm) and, therefore, with larger volume of tissue being reversibly electroporated, resulted in larger amount of DNA contributing to transfection efficiency. However, the predicted transfection efficiency is still significantly lower than in the case of finger or needle electrodes. This indicates, that the conductivity of stratum corneum is probably higher than currently estimated. A similar observation was recently reported in [36]. It is also important to emphasize that numerical model assumes uniform distribution of LTRs, which is probably a simplification, since electric field distribution is not uniform.

Incomplete knowledge of the stratum corneum conductivity is probably the main reason also for inconsistencies regarding the optimization of different HV–LV pulse combinations. According to the model, the amplitude of HV pulse is more important for electrotransfer efficiency than the amplitude of LV pulse, contrary to experimental results of luciferase expression. This inconsistency may arise from a difference in the LTR density, which is associated with the HV pulse amplitude. However, this parameter was not studied more in detail, since introducing additional LTRs to the geometry would prolong the already time consuming simulations. Also, other parameters of which importance remains to be investigated, in addition to the parameters mentioned at the beginning of the discussion (the delivery site, pH changes, plasmid concentration), should not be overlooked. For instance, the amount of conductive gel and precise administration of a plasmid volume, to name additional few experimental variables that need to be controlled or their influence evaluated.

## Conclusion

A three dimensional numerical model was built describing gene electrotransfer to skin. The model simulates the electrophoretic movement of plasmid DNA through the nonuniform distribution of electric field and thus, enables to assess the volume of transfected cells. However, simulation results failed to explain the differences between different types of electrodes and pulse combinations, observed in experiments reported previously [17]. The increase in electrical conductivity of the stratum corneum due to electroporation was identified as one of the critical parameters of the model. Therefore, a better understanding of stratum corneum electroporation as well as other variables and processes involved in gene electrotransfer is required to obtain more accurate results.
